# The fusion band in V1: a simple ECG guide to optimal resynchronization? An echocardiographic case report

**DOI:** 10.1186/1476-7120-3-29

**Published:** 2005-09-16

**Authors:** Lorella Gianfranchi, Katia Bettiol, Federico Pacchioni, Giorgio Corbucci, Paolo Alboni

**Affiliations:** 1 Division of Cardiology, Ospedale Civile, Cento Via Vicini 2 Cento (Fe) Italy

## Abstract

**Background:**

Patients with left bundle branch block have a preserved right bundle branch conduction and the efficacy of left ventricular pacing could be explained with the fusion between artificial pulse delivered in the left lateral wall and the spontaneous right ventricular activation. Moreover, the efficacy of left ventricular pacing could be enhanced with an optimal timing between the spontaneous right ventricular activation and the left ventricular pulse.

**Case presentation:**

We evaluated a patient (male, 47 yrs) with surgically corrected mitral regurgitation, sinus rhythm and left bundle branch block, heart failure (NYHA class III) despite medical therapy and low ejection fraction (25%): he was implanted with a biventricular device.

We programmed ventricular pacing only through the left ventricular lead.

We defined what we called electrocardiographic "fusion band" as follow: programming OFF the stimulator, we recorded the native electrocardiogram and measured, through the device, the intrinsic atrioventricular interval. Then, atrioventricular interval was progressively shortened by steps of 20 ms down to 100 ms. Twelve leads electrocardiogram was recorded at each step. The fusion band is the range of AV intervals at which surface electrocardiogram (mainly in V1 lead) presents an intermediate morphology between the native left bundle branch block (upper limit of the band) and the fully paced right bundle branch block (lower limit).

The patient underwent echocardiographic examination at each atrioventricular interval chosen inside the fusion band. The following parameters were evaluated: ejection fraction, diastolic filling time, E wave deceleration time, aortic velocity time integral and myocardial performance index.

All the echocardiographic parameters showed an improvement inside the fusion band, with a "plateau" behaviour. As the fusion band in this patient ranged from an atrioventricular delay of 200 ms to an atrioventricular delay of 120 ms, we chose an intermediate atrioventricular delay of 160 ms, presuming that this might guarantee the persistence of fusion even during any possible physiological (autonomic, effort) atrioventricular conduction variation.

**Conclusion:**

In this heart failure patient with left bundle branch block, tailoring of the atrioventricular interval resynchronized myocardial contraction with left ventricular pacing alone, utilizing a sensed right atrial activity and the surface electrocardiographic pattern.

## Background

Patients with heart failure (HF), left ventricular dysfunction and left bundle branch block (LBBB) can be treated with cardiac resynchronization therapy (CRT) [1], which can be obtained with both biventricular pacing or left ventricular (LV) pacing. Good results have been reported with the two types of pacing [2-12].

Patients with LBBB generally have a preserved right bundle branch conduction and the efficacy of LV pacing can be explained with a fusion between the artificial pacing pulse delivered in the left lateral wall and the spontaneous right ventricular (RV) activation. Moreover, the efficacy of LV pacing could be enhanced with an optimal timing between the spontaneous RV activation and the LV pulse [13-15].

Patients with sinus rhythm (SR), HF, left ventricular dysfunction and LBBB can provide a specific trigger for a good timing of ventricular stimulation, namely atrial activity, sensed or paced. In addition, as the atrioventricular (AV) interval can be programmed, the morphology of the QRS complex consequently varies, showing different degrees of fusion. In this context electrocardiogram (ECG) could be used as a marker reflecting the mechanical synchrony of the ventricles.

This case report suggests that a properly timed fusion between the spontaneous RV activation and LV pacing corresponds to a good hemodynamic response of the heart and, therefore, the optimal timing of LV pacing could be determined simply by programming the AV interval on the basis of surface ECG pattern.

## Case presentation

We evaluated a patient (male, 47 yrs) with surgically corrected mitral regurgitation, HF (NYHA class III), SR, LBBB and left ventricular dysfunction (ejection fraction 25%) despite optimal medical therapy; he was implanted with a biventricular device with independent ventricular outputs (Contak Renewal ICD-Guidant).

We programmed ventricular pacing only through the LV lead. The device was tracked by the spontaneous atrial activity because of a preserved sinus node function.

We defined what we called electrocardiographic "fusion band" as follows: the stimulator was first programmed OFF to assess the basal ECG of the patient and the intrinsic AV interval, namely the interval between atrial and RV sensing measured through the implanted device, was measured as well. Then, AV interval was progressively shortened by steps of 20 ms down to the lower limit of 80 ms. Twelve leads ECG was recorded at each step. The fusion band is the range of AV intervals at which surface ECG (mainly V1 lead) shows an intermediate morphology between the native LBBB pattern (upper limit of the band) and the fully paced right bundle brunch block (RBBB) pattern (lower limit).

**Figure 1:**
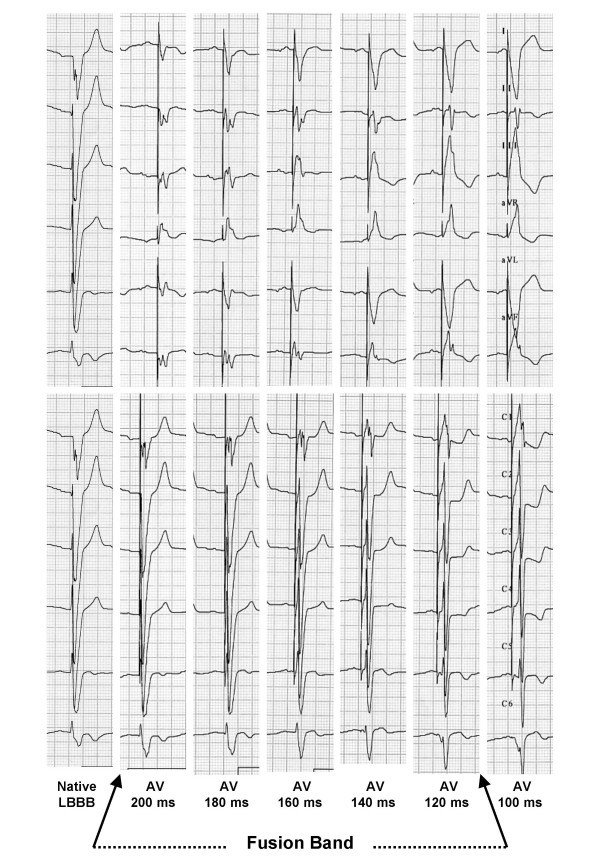
**ECG morphology at different AV intervals. **Different ECG morphology corresponding to each programmed AV interval, including the basal ECG of the patient without ventricular pacing. With the progressive shortening of the programmed AV interval, QRS morphology (mainly in V1 lead) changes from LBBB to a RBBB-like trace at the shortest AV interval, corresponding to a complete capture of both ventricles by LV pacing. Different degrees of ventricular fusion between RV spontaneous activation and LV pacing can be seen for AV intervals programmed in the range 200–120 ms.

In order to investigate the "fusion band", we chose the following AV values: 200, 180, 160, 140, 120 ms (figure 1).

The patient underwent echocardiographic examination at each AV interval. The following parameters were evaluated: ejection fraction (EF%), diastolic filling time (DFT), E wave deceleration time (DT), aortic velocity time integral (VTI) and myocardial performance index (MPI).

**Figure 2:**
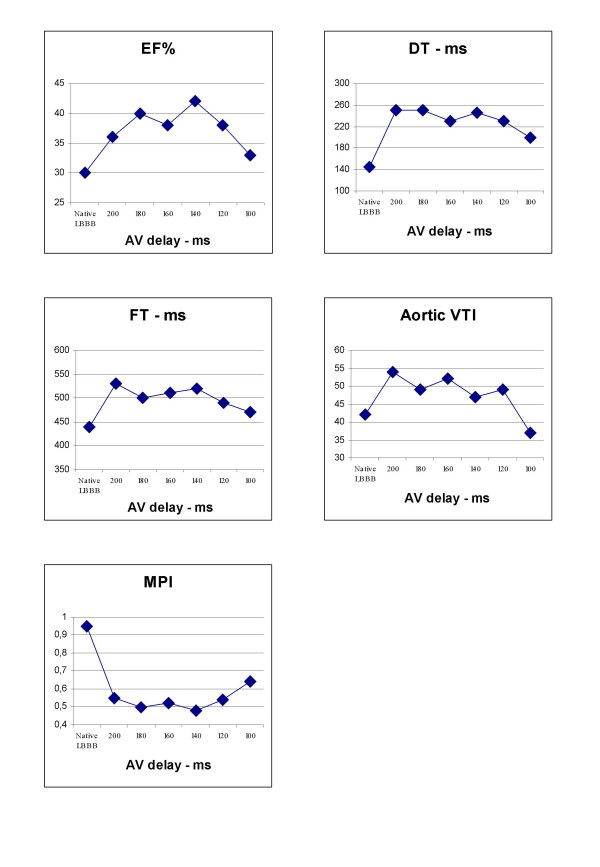
**Echocardiographic parameters at each step of AV interval. **Ejection fraction (EF%), diastolic filling time (DFT), E wave deceleration time (DT), Aortic Velocity Time Integral (Aortic VTI), Myocardial performance index (MPI).

All the echocardiographic parameters showed an improvement at each programmed AV interval with a "plateau" behavior. Within an AV interval of 200 and 120 ms we choose to program the AV interval at an intermediate value of 160 ms (figure 2).

## Discussion

In this patient LV pacing seems to produce an improvement of the echocardiographic parameters at every AV interval inside the fusion band. It is not possible, however, to identify an AV value at which there is a full concordance of all the parameters. As the band of fusion ranges from an AV interval of 200 to 120 ms, we chose an intermediate AV value of 160 ms supposing that this might enable the persistence of fusion even during any possible physiological (autonomic, effort, etc) AV conduction variation. Data from literature support this hypothesis [9] since LV pacing alone seems effective in the mid-term in this patient population. This suggests that the acutely tailored AV interval is a valid option also during the spontaneous variation of the AV conduction. For this reason, LV pacing does not seem to be a good option in patients with AV block, due to the lack of spontaneous RV activation.

The improvement of every echocardiographic parameter inside the fusion band, as compared to the basal conditions, shows a plateau pattern. This has to be confirmed by a large-scale trial. We hypothesize that our method could be applied in the patients undergoing CRT, representing a simple and effective way to program the proper AV interval using only surface and intracardiac ECG analysis.

If further studies will confirm this hypothesis, the programming of the devices would be simplified and made faster without any loss in effectiveness. Moreover this method saves the energy of RV pacing.

The hypothesis of tailoring myocardial resynchronization with LV pacing alone from ECG criteria seems attractive and LV pacing could be proposed as a valid option in patients with sinus rhythm. This approach does not necessarily exclude the presence of RV lead as only patients with LBBB and stable sinus rhythm may do without RV pacing. In fact the possibility of new development of atrial fibrillation in HF patient must be considered: in this case biventricular pacing may be useful as during atrial fibrillation episodes fusion is not constantly present, due to the loss of the reference a trial trigger for LV pacing.

Moreover, patients with indication to implantable defibrillators need RV lead for the appropriate sensing and defibrillation of the arrhythmias.

## Conclusion

Our HF patient with SR and LBBB showed an improvement of echocardiographic variables during the ECG "fusion band" obtained with LV pacing. This simple method, only based on ECG criteria, needs to be tested on a large group of patients.

## Competing interests

The author(s) declare that they have no competing interests.

## Authors' contributions

All the authors contributed to the definition of the proposed method. The paper has been written by L.G. The draft has been discussed by all the authors and the final version approved.
